# The Effect of Endophytic Bacteria *Bacillus subtilis* and Salicylic Acid on Some Resistance and Quality Traits of Stored *Solanum tuberosum* L. Tubers Infected with Fusarium Dry Rot

**DOI:** 10.3390/plants9060738

**Published:** 2020-06-11

**Authors:** Oksana Lastochkina, Liudmila Pusenkova, Darya Garshina, Ruslan Yuldashev, Irina Shpirnaya, Cemal Kasnak, Recep Palamutoglu, Ildar Mardanshin, Svetlana Garipova, Mohammadhadi Sobhani, Sasan Aliniaeifard

**Affiliations:** 1 Bashkir Research Institute of Agriculture—Subdivision of the Ufa Federal Research Centre of the Russian Academy of Sciences, 450059 Ufa, Russia; l.pusenkova@mail.ru (L.P.); dariya.greatfire@mail.ru (D.G.); ildar.mardanshin1966@yandex.ru (I.M.); garipovasvetlana@gmail.com (S.G.); 2 Institute of Biochemistry and Genetics—Subdivision of the Ufa Federal Research Centre of the Russian Academy of Sciences, 450054 Ufa, Russia; yuldashevra@gmail.com; 3 Department of Biology, Bashkir State University, 450076 Ufa, Russia; i-shia@yandex.ru; 4 Department of Nutrition and Dietetics, Health Sciences Faculty, Afyonkarahisar Health Sciences University, 2078 Afyonkarahisar, Turkey; ckasnak@gmail.com (C.K.); receppalamutoglu@hotmail.com (R.P.); 5 Photosynthesis Laboratory, Aburaihan Campus, University of Tehran, Tehran 3391653775, Iran; sobhanimoeidgirizli@gmail.com (M.S.); aliniaeifard@ut.ac.ir (S.A.)

**Keywords:** endophytic *Bacillus subtilis*, salicylic acid, potatoes, storage, *Fusarium oxysporum*, resistance, hydrolytic enzymes, glycoalkaloids, quality

## Abstract

The effect of endophytic *Bacillus subtilis* (strains 10-4, 26D) and their compositions with salicylic acid (SA) on some resistance and quality traits of stored potatoes infected with Fusarium dry rot were studied. The experiments were carried out on hydroponically grown *Solanum tuberosum* L. tubers that were infected before storage with *Fusarium oxysporum* and coated with *B. subtilis* 10-4, 26D with and without exogenous SA, and then stored for six months. It has been shown that 10-4, 26D, 10-4 + SA, and 26D + SA reduced in different levels (up to 30–50%) the incidence of *F. oxysporum*-caused dry rot (with the highest effect for 10-4 + SA). SA notably enhanced the positive effect of 10-4, while for 26D, such an effect was not observed. All of the tested treatments increased amylase (AMY) and AMY inhibitors activity in infected tubers, while decreased *Fusarium*-induced protease activity (except in the case of 10-4 + SA, which promoted a slight increase) was revealed. 10-4, 26D, and their compositions with SA decreased (in different degrees) the pathogen-caused lipid peroxidation, proline, and reducing sugars accumulation in potatoes after long-term storage. It was also discovered 10-4 and 26D, regardless of SA presence, decrease pathogen-induced glycoalkaloids α-Solanine and α-Chaconine accumulation and preserved increased levels of starch and total dry matter in infected stored potatoes. The findings indicate endophytic *B. subtilis* and its compositions with SA is a promising eco-friendly and bio-safe approach to cope with postharvest decays of potato during long-term storage; however, when developing preparations-compositions it should take into account the strain-dependent manner of *B. subtilis* action together with SA.

## 1. Introduction

Potato (*Solanum tuberosum* L.) is a valuable food crop with great importance in ensuring food security worldwide [1]. One of the most acute problems in modern agriculture and food industries is the loss of potato tubers (about 50–60% of the total harvest) during storage from diseases [2]. Fusarium dry rot of potato that is caused by *Fusarium* spp. is a major devastating disease that causes postharvest rotting in storage and seed tubers decay [3,4]. About thirteen different *Fusarium* spp. have been identified as agents that are responsible for potato dry rot worldwide. Among them, the most common species are *F. oxysporum*, *F. solani*, *F. sambucinum*, *F. avenaceum*, *F. culmorum*, *F. acuminatum*, *F. equiseti*, and *F. crookwellense* [4,5]. Pathogens often infect plants and live in their tissues without any typical disease symptoms at growing time and are not always visible at the time of harvest, but may rapidly appear after harvesting and become the major decay factor. Initially, lesions appear as brown or black spots on the surface of the tuber. Lesions later form large, hollow cavities. Often, the affected areas appear wrinkled on the surface of the tuber with numerous white bundles of mycelium. Losses from *Fusarium*-associated dry rot has been estimated to reduce up to 25% crop establishment by affecting the development of potato sprouts and more than 60% of tubers can be infected during long-term storage [3,5]. Moreover, *Fusarium* spp. might contaminate food products with mycotoxins (of which the most common groups are trichothecenes, zearalenones, fumonisins, enniatins, moniliformin, and beauvericin, etc.), which threatens animal health and can cause a wide range of infections of humans [6,7,8]. Potatoes may be infected by pathogens any time during the growing season or through wounds inflicted during harvesting, sorting, transportation, and handling for storage [9]. Traditionally applied chemicals for control of postharvest decay are hazardous to human, animal, and environmental health due to the toxicological risk, and its application is prohibited in many countries [10]. Environmentally friendly and safe approaches, which might induce the natural defense mechanisms of the plant organisms against diseases, are important alternative strategies [2,9,10]. The most effective approaches to control dry rot are the utilization of resistant potato cultivars, although better handling of potatoes at harvest and during postharvest operations would sensibly reduce the impact of such diseases as well [2,9].

Beneficial antagonistic bacteria *Bacillus subtilis* (with a well-known role in plant growth promotion and anti-stress physiological programs induction), generally recognized as safe microorganisms (GRAS) to use in the food industry, are considered a bio-active and eco-friendly agent for controlling postharvest decay [9,10,11]. To date, numerous examples of successful *B. subtilis* application in the control of various postharvest-emerged pathogens of different fruits/vegetables during handling, transportation, and storage have been described in the literature [9,12,13,14]. Their mechanisms of action are still largely unclear; however, it is suggested that they include competition for space/nutrients with pathogens, production of various bio-active substances with antibiotic activity and cell wall-degrading compounds, and the induction of systemic resistance in whole host-plant organisms [9,11,15]. These features, together with the sporulation ability of *B. subtilis*, make them ideal for the development of commercial bioproducts [9]. With that, *Bacillus* efficiency might depend on various factors, including strain characteristics (epiphytes or endophytes), application methods (before or after harvest/storage), type of pathogens/hosts, etc. [9,16]. Additionally, there is interest in the co-application of *B. subtilis* alongside other biological agents in an integrated vision of disease management due to the lack of individual strains with broad spectrum activity against a wide range of pathogens. Salicylic acid (SA) is a safe signaling molecule that can be used for the development of complex bioproducts with wide spectrums of action in both preharvest and postharvest strategies, while having high commercial potential for enhancing nutritional quality along with the extension of the shelf-life of fruits/vegetables, including potato [9]. Numerous studies have indicated the potential of SA postharvest application for reducing chilling injury and decay, delaying ripening, and enhancing the health benefits of fruit and vegetable consumption by increasing antioxidant capacity [17]. However, there is little information regarding the influence of the joint application of endophytic *B. subtilis* with SA on plant growth, development, and postharvest physiology of healthy and pathogens-infected potatoes during long-time storage [16,18,19]. In our recent work, we generated data about endophytic *B. subtilis* (strains 10-4, 26D), alone or in compositions with SA, increasing the resistance of potato tubers to *Phytophthora infestans* and *Fusarium oxysporum* during long-time storage, which was accompanied with prolonging shelf-life of potatoes and preserving their appearance [16]. The findings suggest *B. subtilis* decrease as disease incidence in potato due to colonizing internal tissues and protect cells inside against pathogen development and stress-induced cell damages, therewith, controlling aging processes [16]. However, the mechanisms underlying *B. subtilis* actions both alone and in compositions with SA on potato under postharvest pathogen’s infection are largely unknown and they require further detailed investigations to fully realize their potential in agricultural/food industries.

The main mode of action driving the antifungal effect of *B. subtilis* might be due to the disruption of the cellular membrane structure of pathogens by hydrolases, capable of destroying the structural polysaccharides of the fungal cell wall and lysing the hyphae of fungi [9,20]. A correlation between antagonistic activity to various pathogenic fungi and the synthesis of hydrolases, such as amylases (AMY) and proteases (PRO), by a number of bacteria has been established [9,21]. The protective response of plants is an increase in the content of hydrolase inhibitors, which make a significant contribution to the regulation of the activity of hydrolytic enzymes by suppressing the activity of their own native enzymes, and those from pathogenic fungi and bacteria [20,22,23]. Previously, on model plants of potato and sugar beet, we showed that *B. subtilis*-based bioproducts promoted PRO inhibitors synthesis and protected growing plants from the penetration/development of pathogenic microorganisms [18,24,25]. With that, it is an interest in the character of changing in hydrolytic enzymes and their inhibitors in harvested potato tubers that are treated with *B. subtilis* upon pathogen infection during long-term storage.

As many other stresses, pathogens attack leads to the synthesis of a range of defense compounds in plant cells to protect them from damages [26,27,28]. As an important biochemical marker for the formation of plants resistance can be the accumulation of malondialdehyde (MDA) (the final product of stress-caused lipid peroxidation) and a multifunctional stress metabolite proline, which acts as an antioxidant, osmolyte [29], and a low molecular weight chaperone [23], which is involved in maintaining the native structure of enzymes [29,30]. Many studies reported an increase in proline content in plants in response to different stresses and its importance for plant survival in extreme situations [29,30]. The formation of potatoes resistance can also be assayed by the changes in reducing sugars (RS), total dry matter (TDM), and starch contents in healthy and pathogen-infected stored tubers [31,32], which also largely determine the consumer and table qualities of potatoes and uses to judge the best further options for products processing [31,33]. An increase in the content of RS and starch decomposition into sugar in potato tubers during storage negatively affects the culinary qualities (during heat treatment, the flesh darkens, the taste worsens, and becomes sweet-starchy-viscous) [31,33]. Tubers with higher TDM and starch concentration exhibited the highest resistance to mechanical impacts [34]. Under unfavorable storage conditions (i.e. pathogen attack), normal metabolic pathways change in potato, which leads to the synthesis of abnormal amounts of numerous novel compounds and secondary metabolites, including glycoalkaloids (GA) (α-Solanine and α-Chaconine are 95% of the total GA), which is believed are associated with antimicrobial, antifungal activities [35,36], and play an important role in the disease resistance of tubers [37]. With that, elevated GA levels in potatoes (200 mg/kg of tubers are considered as critical) are toxic compounds that pose a serious potential hazard to humans [38,39]. The highest accumulation of toxic GA levels in potatoes typically occurs in the storage period. However, the correlation between the content of GA in potatoes during storage and under conditions of infection by *F. oxysporum*, and the use of endophytic bacteria *B. subtilis* and *B. subtilis* + SA in the available literature has not been established at the moment of the beginning of our work.

In this work, we investigated the effect of endophytic *B. subtilis* (strains 10-4, 26D) and compositions of *B. subtilis* (10-4, 26D) with SA on the development of Fusarium dry rot, the activity of hydrolytic enzymes and their inhibitors, the level of lipid peroxidation and proline content (as markers of oxidative and osmotic stresses development, respectively), reducing sugars, starch, total dry matter, and glycoalkaloids accumulation in healthy (non-infected) and *F. oxysporum*-infected potato tubers after long-term storage.

## 2. Results

### 2.1. Supression of F. Oxysporum Development in Stored Potato Tubers by Endophytic B. Subtilis (10-4, 26D) and B. Subtilis (10-4, 26D) + SA

**Figure 1:**
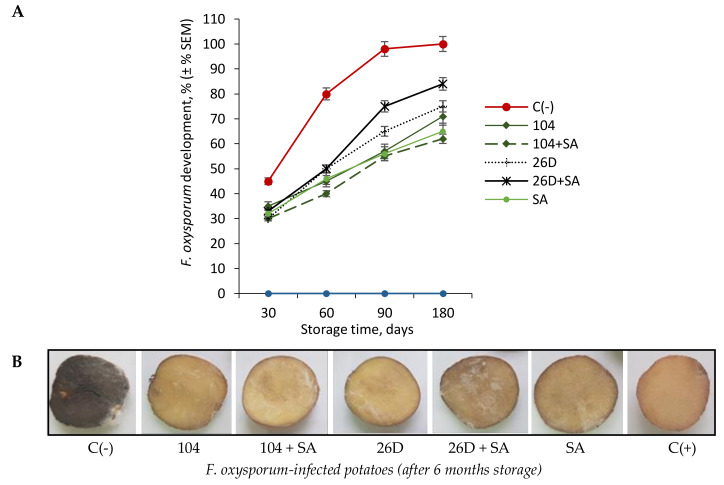
Effect of *B. subtilis* 10-4 (104), *B. subtilis* 26D (26D), *B. subtilis* 10-4 + salicylic acid (SA) (104 + SA), and *B. subtilis* 26D + SA (26D + SA) on *F. oxysporum* development in potatoes during long-time storage for six months (**A**) and pictures of tubers stored six months after infestation with *F. oxysporum* and coated with *B. subtilis* strains 10-4, 26D, and their compositions with SA (**B**). For each treatment was used 30 mini-tubers in three replicates (± SEM). C(-)—negative control tubers infected before storage with *F. oxysporum*; 104—tubers infected with *F. oxysporum* and treated with *B. subtilis* 10-4; 104 + SA—tubers infected with *F. oxysporum* and treated with composition *B. subtilis* 10-4 + SA; 26D—tubers infected with *F. oxysporum* and treated with *B. subtilis* 26D; *B. subtilis* 26D + SA—tubers infected with *F. oxysporum* and treated with *B. subtilis* 26D + SA, SA—tubers infected before storage with *F. oxysporum* and treated with SA; C(+)—positive control tubers without infection and treatments.

The artificial infection of potato tubers by *F. oxysporum* overtime led to a gradual increase in symptoms of *Fusarium* dry rot, reaching 100% by six months of storage was found (Figure 1A,B). The bacterization of tubers immediately before storage with pre-established [16] concentrations of *B. subtilis* (10-4, 26D), in compositions with and without SA, resulted in the reduced intensity of *F. oxysporum* development, manifested as a 30–50% decrease in the area of lesions after long-time storage (Figure 1A,B). The most positive effect in the suppression of *F. oxysporum* development in stored tubers treated with 10-4 *+* SA was observed.

**Figure 2:**
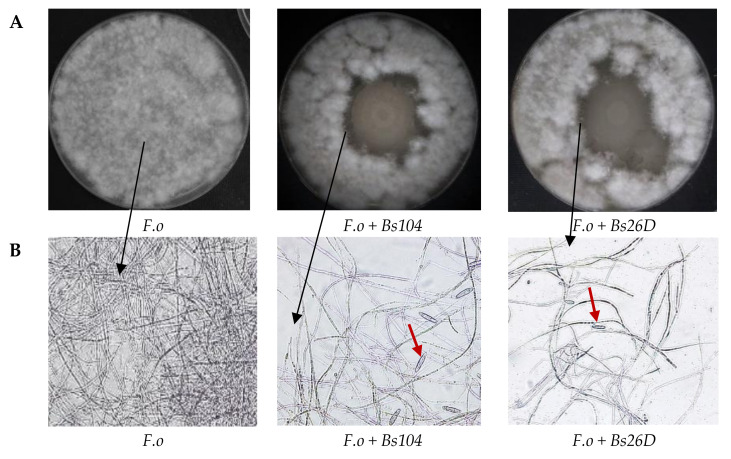
In vitro antagonistic activity of tested *B. subtilis* 10-4 (Bs104) and *B. subtilis* 26D (Bs26D) against the phytopathogenic fungus *F. oxysporum* (F.o) (**A**) and microscopic visualizations of the *F. oxysporum* fungal growth and morphology in the absence and presence of *B. subtilis* 10-4 and 26D (**B**). The observation was done using a scanning electron microscope Biozero BZ-8100E (Keyence Co., Osaka, Japan). *F.o*—*F. oxysporum*; *Bs*—*B. subtilis*. Red arrows mean macroconidia produced by *F. oxysporum*.

In vitro studies also showed that *B. subtilis* 10-4 and 26D have antagonistic activity against the phytopathogenic fungus *F. oxysporum* (Figure 2A). The microscopic observation of the *F. oxysporum* fungal mycelia clearly revealed morphological variations. The structure of the *F. oxysporum* mycelia was well organized in the absence of the bacterial culture medium (Figure 2B), while numerous gaps of mycelia appeared and macroconidia were produced in the presence of the culture medium of strains 10-4 and 26D (Figure 2B).

### 2.2. Effect of Endophytic B. subtilis 10-4, 26D and Their Compositions with SA on Activity of Hydrolitic Enzymes Proteases (PRO), Amylases (AMY) and Their Inhibitors in Stored Non-Infected and F. oxysporum-Infected Tubers

**Figure 3:**
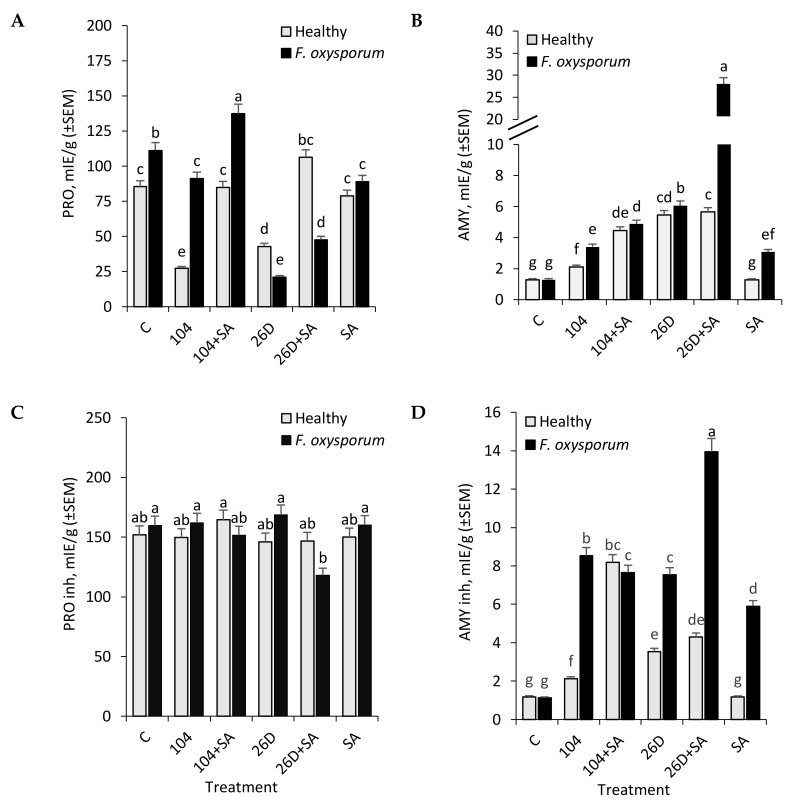
Effect of endophytic bacteria *B. subtilis* 10-4 (104) and *B. subtilis* 26D (26D), individually and in compositions with SA (104 + SA, 26D + SA), on activities of hydrolytic enzymes protease (PRO) (**A**), amylase (AMY) (**B**) and inhibitors of protease (PRO inh) (**C**) and inhibitors of amylase (AMY inh) (**D**) in *F. oxysporum*-infected (*F. oxysporum*) and non-infected (Healthy) potato tubers during storage (time of storage six months). The bars are the means of three repetitions  ±  SEM. Different letters indicate a significant difference between the means at the probability level of *p*  < 0.05.

It was found that *F. oxysporum* increased (up to 1.3 times) the activity of proteases (PRO) in potato tubers after long-time (six months) storage (Figure 3A), while there were no changes in the activity of amylase (AMY) as compared to non-infected (healthy) control (Figure 3B). At the same time, applications of 10-4, 26D, 26D + SA, and SA decreased (by 1.2–5.3 times) pathogen-caused increase in PRO activity (except 10-4 + SA, where the additional increase was observed) (Figure 3A). In healthy tubers, 10-4 + SA and SA had no effect on PRO activity, 10-4 and 26D decreased (by 3.1 and 2 times, respectively), and for 26D + SA, some even increase (up to 1.2 times) was revealed. In tubers that were infected with *F. oxysporum* the activity of PRO inhibitors was comparable to control due to only a slight increase (from 151.8 mIE/g in healthy control to 159.6 mIE/g in *Fusarium*-infected ones) were observed. The application of *B. subtilis*, SA, and their compositions also had no significant effect on PRO inhibitors activity on both healthy and *Fusarium*-infected tubers (Figure 3C). However, for 10-4 + SA and 26D + SA (upon infection with *F. oxysporum*), a slight decrease in the activity of PRO inhibitors was observed, and a slight increase upon 10-4 + SA treatment in healthy tubers.

With that, in response to treatment with 10-4, 26D individually and in compositions with SA, there was a significant increase in the activity of AMY both in healthy (ranging from 1.6 to 4.4 times) and in *F. oxysporum*-infected (from 2.6 to 21.8 times) tubers during storage (Figure 3B) (with the notable increase (up to 21.8 times in comparison to control) in tubers treated with 26D + SA upon *Fusarium* infection). In healthy tubers only treated with SA, there were no differences in the activity of AMY in comparison to control, while in infected tubers some increase (up to 2.9 times) was detected (Figure 3B). The activity of AMY inhibitors also increased under the influence of *B. subtilis* (10-4, 26D) and *B. subtilis* (10-4, 26D) + SA applications both in healthy and especially *F. oxysporum* infected tubers (Figure 3D). In particular, the activity of AMY inhibitors in non-infected (healthy) tubers was 1.18 mIE/g on fresh weight of tubers (control without bacterial treatments), 2.12 mIE/g (for 10-4), 8.18 mIE/g (for 10-4 + SA), 3.53 mIE/g (for 26D), and 4.29 mIE/g (for 26D + SA) (Figure 3D). Meanwhile, in tubers that were infected by *F. oxysporum*, there was no significant difference from non-infected control in AMY inhibitors activity, while, in tubers treated with *B. subtilis* (10-4, 26D) and their compositions with SA, *F. oxysporum* infection correlated with an increase in the activity of AMY inhibitors ranging from 7.53 mIE/g (for 26D), 7.65 mIE/g (10-4 + SA), 8.53 mIE/g (10-4), to 16.94 mIE/g (for 26D + SA).

### 2.3. Effect of Endophytic B. Subtilis 10-4, 26D and Their Compositions with SA on Malondialdehyde (MDA) and Proline Contents (as Markers of Oxidative and Osmotic Stresses Development) in Stored Non-Infected and F. oxysporum-Infected Tubers

**Figure 4:**
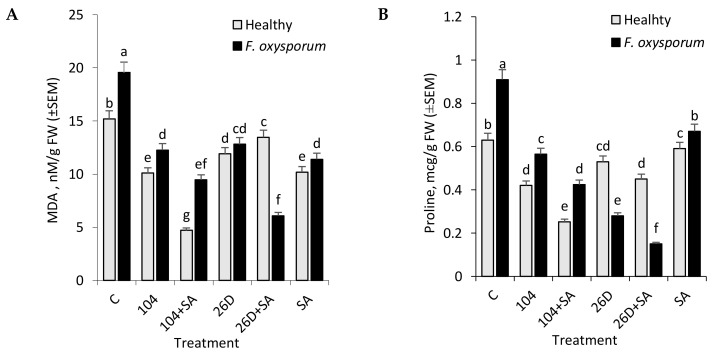
Effect of *B. subtilis* 10-4 (104), *B. subtilis* 26D (26D), *B. subtilis* 104 + salicylic acid (SA) (104 + SA), and *B. subtilis* 26D + SA (26D + SA) on the content of malondialdehyde (MDA) (**A**) and proline (**B**) in *F. oxysporum*-infected (*F. oxysporum*) and non-infected (Healthy) stored potato tubers (time of storage six months). The bars are the means of three repetitions ± SEM. Different letters indicate a significant difference between the means at the probability level of *p* < 0.05.

*F. oxysporum* leads to an increase in MDA level and Pro (biomarkers of oxidative and osmotic stresses development) concentrations in potato tubers during long-term storage (Figure 4A,B). The application of *B. subtilis* 10-4, *B. subtilis* 26D, *B. subtilis* 10-4 + SA, and *B. subtilis* 26D + SA decreased (in different degrees) the level of *F. oxysporum*-caused accumulations of MDA (by 1.5–3.2 times) and Pro (by 1.4–6 times) with the most notable effects being when using the composition of 26D + SA. Application of SA also decreased *Fusarium*-caused lipid peroxidation and Pro accumulation and its action were comparable with actions of strain 10-4 and 26D. At the same time, the application of SA together with strain 10-4 and 26D showed the different manner of changes in MDA and Pro. Particularly, when SA and 10-4 were used together, the content of MDA and Pro was lower than in variants with their individual application (Figure 4A,B). In healthy tubers (non-infected with *F. oxysporum*), the application of 10-4, 26D individually and in compositions with SA reduced MDA and Pro levels (in comparison with non-bacterized control) were observed. Accordingly, in tubers that were treated with 10-4 and 26D, the levels of MDA were lower by 1.5 and 1.3 times (in comparison to control), respectively. While, when 10-4 and 26D were used in compositions with SA, the level of MDA reduced by 3.2 and 1.1 times (Figure 4A). The same manner of changes in Pro content was observed in healthy tubers that were treated with 10-4, 26D, and their compositions with SA (Figure 4B). Thus, it was found that 10-4, 26D, individually and in compositions with SA reduced (in different levels) *Fusarium*-caused increase in lipid peroxidation (MDA) and Pro content in stored tubers with the highest effect for 26D + SA, while, in healthy (non-infected with *Fusarium*) tubers, the highest effect was observed for 10-4 + SA.

### 2.4. Influence of Endophytic B. Subtilis (10-4, 26D) and B. Subtilis (10-4, 26D) + SA on the Content of Reducing Sugars (RS), Starch and Total Dry Matter (TDM) in Non-Infected, and F. oxysporum-Infected Stored Tubers

**Figure 5:**
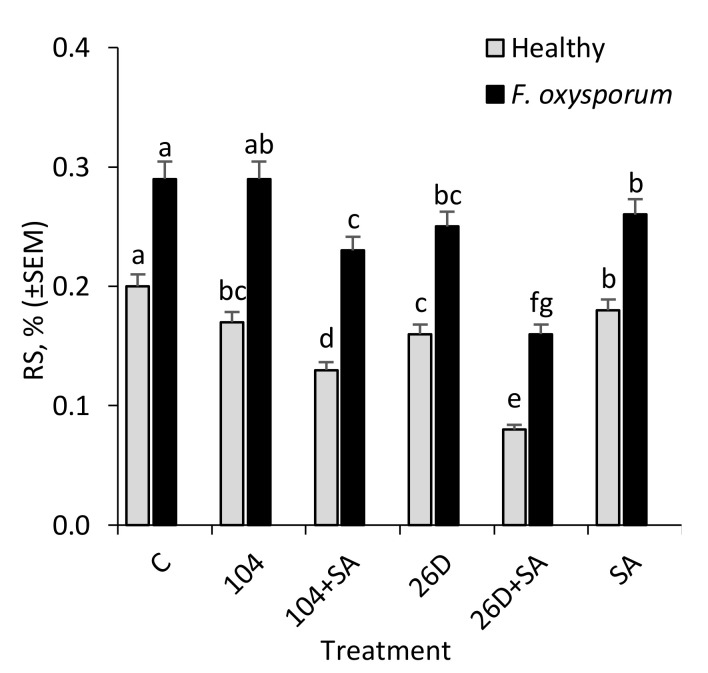
Changes in the contents of reducing sugars (RS) in non-infected (Healthy) and infected with the phytopathogenic fungus *F. oxysporum* (*F. oxysporum*) potato tubers under the influence of treatments with *B. subtilis* 10-4 (104), *B. subtilis* 10-4 + SA (104 + SA), *B. subtilis* 26D (26D) and *B. subtilis* 26D + SA (26D + SA) after six months storage. The bars are the means of three repetitions ± SEM. Different letters indicate a significant difference between the means at the probability level of *p  *<  0.05.

**Figure 6:**
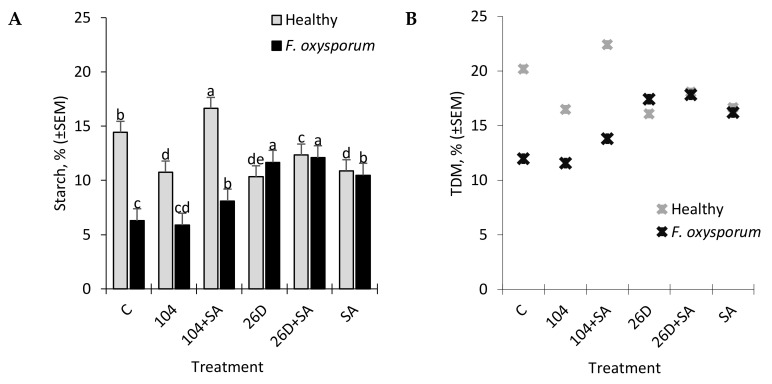
Changes in the contents of starch (**A**) and total dry matter (TDM) (**B**) in non-infected (Healthy) and infected with the phytopathogenic fungus *F. oxysporum* (*F. oxysporum*) potato tubers under the influence of treatments with *B. subtilis* 10-4 (104), *B. subtilis* 10-4 + SA (104 + SA), *B. subtilis* 26D (26D), and *B. subtilis* 26D + SA (26D + SA) after six months storage. The bars are the means of three repetitions ± SEM. Different letters indicate a significant difference between the means at the probability level of *p* <  0.05.

In tubers that were infected with *F. oxysporum* after six months of storage, an increased level of reducing sugars (RS) was detected in comparison with non-infected control ones (Figure 5). It was found that the treatment of potato tubers with *B. subtilis* 10-4, *B. subtilis* 26D, *B. subtilis* 10-4 + SA, and *B. subtilis* 26D + SA contributed to a decrease (in different degrees reaching up to 1,8 times) of *F. oxysporum*-induced RS increase in stored potatoes (Figure 5). In healthy stored tubers *B. subtilis* and *B. subtilis* + SA decreased RS content (up to 1.2–2.5 times) in comparison with the control, which indicated that *B. subtilis* also feeds on sugars, including those that result from pathogen-induced activation of hydrolytic enzymes that break down starch into sugars. Additionally, it was revealed that infestation by *F. oxysporum* results in the reduction of starch content in stored potatoes from 14.4% (in non-infected control) to 6.2% (in infected tubers) (Figure 6A). The application of *B. subtilis* 26D, *B. subtilis* 26D + SA, *B. subtilis* 10-4 + SA prevented such pathogen-effected starch reduction, while, for *B. subtilis* 10-4, there was no significant difference from infected control. In particular, the content of starch ranged from 5.9% (10-4) to 12.1% (26D + SA) (Figure 6A). It should be mentioned that, in cases when *B. subtilis* were applied together with SA, the starch percentage was higher than in variants with the application of *B. subtilis* alone. Interestingly, non-infected (healthy) tubers that were treated before storage with *B. subtilis* 10-4, *B. subtilis* 26D, and *B. subtilis* 26D + SA also exhibited a decrease in starch content (up to 10.3–12.3%) in comparison to control (14.4%), while, for *B. subtilis* 10-4 + SA, in contrast, increased starch percentage (up to 16.6%). The same pattern of change from *B. subtilis* (10-4, 26D) with and without SA were observed for total dry matter (TDM) levels in non-infected (Healthy) and *Fusarium*-infected tubers (Figure 5B). In healthy tubers treated with 10-4, 26D, 26D + SA after long-time storage the level of TDM concentration decreased (up to 16.1–18.1%) in comparison with the control (20.2%), while 10-4 + SA caused an increase (up to 22.5%) (Figure 6B). The increased levels of starch and TDM percentages in healthy tubers treated with *B. subtilis* 10-4 + SA (Figure 6A,B) are most likely to happen due to the trigger of some other additional protective mechanisms by SA in composition with 10-4, while, for the composition 26D + SA, the picture was different.

### 2.5. Effect of Endophytic B. Subtilis 10-4, 26D, and Their Compositions with SA on Content of Glycoalkaloids (GA) in Stored Non-Infected and F. Oxysporum-Infected Tubers

**Figure 7:**
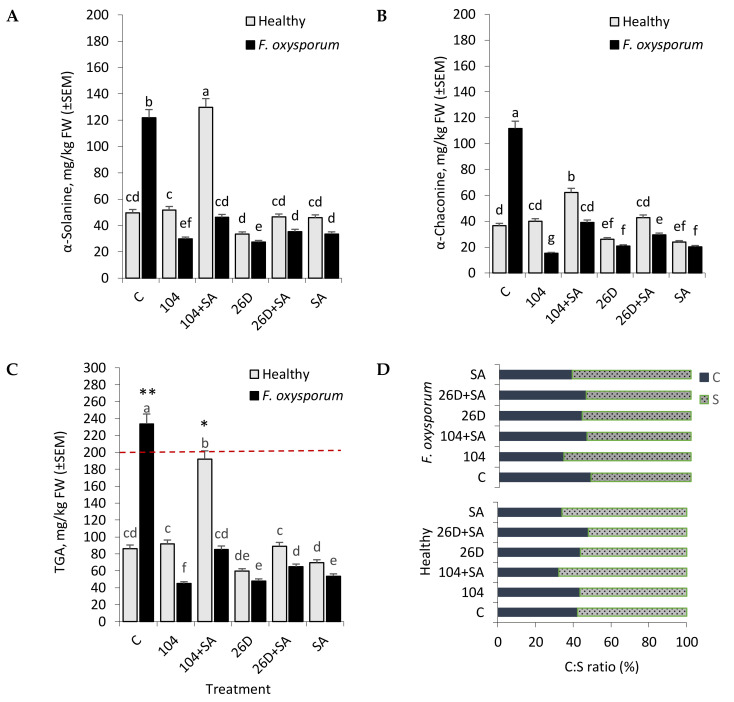
Effect of *B. subtilis* 10-4 (104), *B. subtilis* 10-4 + salicylic acid (SA) (104 + SA), *B. subtilis* 26D (26D), and *B. subtilis* 26D + SA (26D + SA) on the content of glycoalkaloids (GA) ɑ-Solanine (**A**), ɑ-Chaconine (**B**), total GA [TGA] (**C**), and the ratio of α-Chaconine (C) to α-Solanine (S) (C:S ratio) (**D**) in stored non-infected (Healthy) and infected with *F. oxysporum* (*F. oxysporom*) tubers after six months of storage. Data are presented as mean concentrations in milligrams glycoalkaloid per kilogram (mg/kg) fresh weight (FW) of potato tubers. The bars are the means of three repetitions ± SEM. Different letters indicate a significant difference between the means at the probability level of *p*  <  0.05. ** means the value is above the acceptable safe level (200 mg/kg) and * means the value is close to the critical level.

It was discovered that the infestation of tubers with *F. oxysporum* caused an increase in the level of GA, both α-Solanine (from 49.7 mg/kg FW of potatoes in control to 121.9 mg/kg in pathogen-infected) and α-Chaconine (from 36.5 mg/kg in control to 111.7 mg/kg in pathogen-infected) by the sixth months of storage (Figure 7A,B). The use of *B. subtilis* (10-4, 26D) individually and in compositions with SA significantly decreased the level of α-Solanine (Figure 7A) and α-Chaconine (Figure 7B). In non-infected (healthy) tubers, the content of α-Solanine and α-Chaconine had a different profile depending on the variant of treatment (Figure 6A,B). In particular, in variants with the application of 10-4 and 26D + SA, there was no significant difference from control in the content of α-Solanine and α-Chaconine. In contrast, the application of 104 + SA increased (2–2.3 times in comparison with control) both α-Solanine (up to 129.9 mg/kg) and α-Chaconine (up to 62.3 mg/kg) content in healthy tubers. Additionally, the application of 26D resulted in decreased (1.4 times) α-Solanine (Figure 7A) and α-Chaconine (Figure 7B) as compared to the control treatment. In general, upon *F. oxysporum* infection, the content of TGA in six months stored potato tuber reached by 233.7 mg/kg and exceeded the maximum permissible level (200 mg/kg) (Figure 7C). While the application of tested *B. subtilis* and their compositions wits SA notably reduced such *F. oxysporum*-induced GA accumulation.

The proportion of α-Chaconine to α-Solanine (C:S ratio) ranged from 42:58 (in control) to 34:66 (strain 10-4), 46:54 (strain 10-4 + SA), 43:57 (strain 26D), and 48:52 (strain 26D + SA) in fresh non-infected (Healthy) tubers (Figure 7D). In contrast, F. oxysporum-infected stored tubers exhibited a C:S ratio of 48:52 (in control), 44:56 (strain 10-4), 32:68 (strain 10-4 + SA), 44:56 (strain 26D), and 45:55 (strain 26D + SA) (Figure 7D). In general, the observed changes in α-Chaconine and α-Solanine concentration, as well as their proportions in stored healthy and pathogen-infected stored tubers, suggests that their involvement in the formation of tubers’ resistance against F. oxysporum upon the influence of applied B. subtilis alone and in composition with SA simultaneously preserving the quality of infected tubers that regulate the level of studied GA.

## 3. Discussion

The infection of tubers with phytopathogenic fungi *F. oxysporum* resulted in typical disease symptom development, manifested as the form of gray (brown) putrefied depressed spots with a white coating, under which the flesh became dry, rotten, the skin wrinkled, and white spores of the fungus appeared on it, which can dissipate and infect neighboring tubers. This becomes visible after 6 months of storage at a temperature of 4 °C, at which point tubers are completely infected (Figure 1A,B). The bacterization with *B. subtilis* 10-4 and 26D reduced the intensity of fusarium disease development, manifested as a decrease in lesion area or even in their absence (Figure 1A,B). Among the tested treatments, the composition of *B. subtilis* 10-4 + SA most effectively decreased the incidence of dry rot caused by *F. oxysporum* in stored potatoes (Figure 1A,B). Moreover, by the sixth months of storage following fusarium infection, tubers that were treated with 10-4 + SA looked healthier and fresher, while, in variants with *B. subtilis* 26D + SA treatment, the picture was different, and traces of damage were became visible on the tubers (Figure 1B). It can be assumed that the mechanisms of action differ between strains 10-4 and 26D and that in the compositions with SA, the protective effect against *Fusarium* is enhanced in one case (strain 10-4 + SA), and not the other (strain 26D + SA) (Figure 1A,B). This finding allows for the possibility that this effect might be associated with the ability of the strains to produce their own phytohormones [13] and activate different pathways that are responsible for suppressing pathogen development, inhibiting the aging process and prolonging shelf-life of stored products. It is likely that, if strain 10-4 produces SA, then the addition of exogenous SA can enhance its protective effect; if strain 26D produces jasmonic acid (JA), the addition of SA to the composition might have the opposite effect, since JA and SA are antagonists. This assumption, of course, requires further detailed study and confirmation.

The main mode of action driving the antifungal effect of *B. subtilis* might be due to the disruption of the cellular membrane structure of pathogens [9]. Our in vitro experiments showed that *B. subtilis* 10-4 and 26D have antagonistic activity against the phytopathogenic fungus *F. oxysporum* (Figure 2A). The microscopic observation of the *F. oxysporum* fungal mycelia clearly revealed morphological variations. The structure of the *F. oxysporum* mycelia was well organized in the absence of the bacterial culture medium (Figure 2B), while numerous gaps of mycelia appeared and macroconidia were produced in the presence of the culture medium of 10-4 and 26D (Figure 2B). The observed phenomenon of macroconidia production by *F. oxysporum* in the presence of 10-4 and 26D (Figure 2B), obviously, connected with the survival strategy of the pathogen and indicates that they are under adverse conditions and produce macroconidia for species preservation. Demonstrated suppression of *F. oxysporum* development in vivo (Figure 1) and in vitro (Figure 2) by *B. subtilis* 10-4 and 26D might also be connected with the ability of these bacteria to produce compounds with antifungal activity, such as cell wall degrading enzymes, lipopeptides, and hormone-like compounds [9]. Accordingly, it has been shown that *B. subtilis* might produce lipopeptides that are characterized by strong antifungal activity [9,11,15,40,41]. It might be proposed that the observed *in vivo* antifungal effect of *B. subtilis* against *F. oxysporum* has its origin in the destabilization of the plasma membrane’s physiological function and that the initial targets could be the functional components, such as the H + -ATPase, which affects fungal integrity. The antifungal effects of *B. subtilis* (10-4, 26D) treatment may also promote the maintenance of firmness, because it protects the potato tubers against fungal physiology, which can involve the secretion of cell wall-degrading enzymes. Overall, detailed investigations both of the spectrum of metabolites produced by 10-4 and 26D and changes in physiological, biochemical, and molecular defense responses in cells of stored potatoes for understanding the mechanisms underlying interaction in systems «endophytic *B. subtilis*—host plants—pathogens» will be quite interesting.

Significant contribution in the bio-control of pathogens is competition for space and nutrients [9]. Bacterial inoculants take up nutrients faster than pathogens; this can lead to the inhibition of germination of pathogen spores at the wound site [9]. A fundamental strategy for nutrient competition might occur via the direct attachment of antagonistic microorganism to pathogen hyphae or via alternative mechanisms, such as the production of a wide range of biologically active molecules, such as antibiotics, biosurfactants, siderophores, hydrogen cyanide, and hydrolases increase their advantage against pathogens, as they compete for a suitable niche for colonization [9]. The possible mechanisms of the antagonistic function of *B. subtilis* can be attributed to the synthesis of extracellular hydrolases that are capable of destroying the structural polysaccharides of the fungal cell wall and lysing the hyphae of fungi [9,20,21]. A correlation between antagonistic activity to various pathogenic fungi and the synthesis of hydrolases such as proteases, cellulases, xylanases, mannanases, and lipases by several bacteria has been established [9,22]. For example, among mucolytic enzymes of *B. subtilis* strain 739, the most contribution to the lysis of the native mycelium of phytopathogenic fungi *F. culmorum*, *Alternaria alternata*, *Bipolaris sorokiniana*, and *Rhizoctonia solani* showed chitinase, chitosanase, β-1,3-glucanases, and proteases [22]. *B. subtilis* APEC170 and *Paenibacillus polymyxa* APEC136 diminish the symptoms of anthracnose and white rot in harvested apples by the inhibition of mycelial growth of the pathogens, which was attributed to the increased production of chitinase, amylase, and protease by *B. subtilis* APEC170 [14]. In another study, chitosanases and proteases produced by *B. subtilis* V26 enhance harvested tomato resistance (up to 79%) to *Botrytis cinerea*-caused postharvest diseases [9]. Our results showed that the application of *B. subtilis* (10-4, 26D) individually and in compositions with SA increased the activity of AMY in stored potato tubers (Figure 3B). It likely occurs due to bacterial enzymes, rather than potato’s own AMY (in control the activity of AMY is lower), since it is known that the nutrition and development of bacteria require soluble forms of carbohydrates. This might explain the increase in the level of AMY activity during treatment with bacterial preparations. An increase in AMY (during infection) is a factor indicating sensitivity, since starch degradation increases and the pathogen develops more actively on soluble sugars. On the other hand, the activation of AMY can be as a result of the intensification of central metabolism, providing energy and metabolites for the subsequent synthesis of protective compounds (for example, when used only SA, we observe this).

The protective response is an increase in the content of hydrolase inhibitors, which, as a rule, does not occur due to an increase in the concentration of constitutive compounds, but due to the synthesis of new forms of inhibitors [22,42]. Plant protein inhibitors make a significant contribution to the regulation of the activity of hydrolytic enzymes by suppressing the activity of their own native enzymes, and those from pathogenic fungi and bacteria [22,23]. Earlier, on model plants of potato and sugar beet, we showed that the introduction of biological products that are based on B. subtilis promotes the synthesis of PRO inhibitors and protects growing plants from the penetration and development of pathogenic microorganisms [18,24,25]. In our current study, *B. subtilis* 10-4 and 26D had practically no effect or the slight decrease in the activity of PRO inhibitors observed in both non-infected (healthy) and *Fusarium*-infected stored potato tubers (Figure 3C). A slight decrease in the activity of PRO inhibitors was observed for *B. subtilis* 26D + SA (upon infection with *F. oxysporum*), resulting in the plant’s resistance to the action of pathogen enzymes and their distribution in tissues affecting the appearance of tubers (Figure 1B). With that, in response to treatment with *B. subtilis* strains 10-4, 26D, and their compositions with SA, there was a significant increase in the activity of AMY inhibitors in both healthy and *F. oxysporum*-infected tubers during storage (Figure 3D). In tubers that were infected by *F. oxysporum*, there was no significant difference from non-infected control in AMY inhibitors activity, while in tubers treated with *B. subtilis* (10-4, 26D) and their compositions with SA *F. oxysporum* infection correlated with an increase in activity of AMY inhibitors (Figure 3D). These findings indicate that *B. subtilis* (10-4, 26D), alone and in compositions with SA, activates the synthesis of AMY inhibitors, with the highest activation being observed when *B. subtilis* 26D was used in composition with SA. The activity of AMY inhibitors is a resistance factor since it is a regulator of the degree of starch hydrolysis. An increase in the level of inhibitory activity (inhibitors of the commercial *B. subtilis* preparation) is explained by an increase in the expression of *B. subtilis* amylase inhibitors, which is probably due to the active colonization and development of endophytes into tubers (acting as their number (quantitate amount) regulator). In our previous work, we demonstrated the ability of *B. subtilis* intensively to colonize internal tissues of tubers and the ability of SA to enhance this process [16].

One of the earliest responses of plant cells to infection by a pathogen is an oxidative burst—the formation of reactive oxygen species (ROS) (H_2_O_2_, superoxide radicals, etc.), resulting in protective reactions of the plant, including the synthesis of the whole spectrum of protective compounds [24,26,27,28]. In an overall picture, the development of plant protective reactions against pathogen-induced oxidative burst correlates with the degree of accumulation of the final lipid peroxidation product—malondialdehyde (MDA) acting as a marker for oxidative stress [29,30]. As a result of oxidative stress in cells denatured proteins (lipid peroxidation products) accumulate in large quantities that act, not only as primary mediators of stress exposure to the stress factor, but also as inducers of the corresponding protective mechanisms of plant cells. Our experiments showed that *F. oxysporum* leads to an increase in MDA level in tubers during long-time storage, while the use of *B. subtilis* 10-4 and *B. subtilis* 26D, both individually and in compositions with SA, contributed to a reduction of the pathogen-induced increases in lipid peroxidation in stored non-infected (healthy) and infected tubers (Figure 4A). These findings indicate a reduction of oxidative stress in stored tubers under the treatments before storage with *B. subtilis* 10-4, *B. subtilis* 10-4 + SA, *B. subtilis* 26D and *B. subtilis* 26D + SA. These phenomena may be related to the modulation activity of oxidative enzymes under their influence, so that they can control the level of H_2_O_2_, which induces lipid peroxidation.

An important biochemical marker for the formation of resistance can also be the accumulation of proline, a multifunctional stress metabolite of plants that acts as an antioxidant, osmolyte [29], and a low molecular weight chaperone [23], which is involved in maintaining the native structure of enzymes [29]. Many studies reported an increase in proline content in plants in response to the stresses of various nature and its importance as a factor that is important for plant survival in extreme situations [29,30]. Our results show that infection of the potato tubers with *F. oxysporum* led to a significant increase in the proline content in the tubers (Figure 4B). At the same time, *Fusarium*-infected tubers that were treated with *B. subtilis* 10-4 and 26D, both individually and in compositions with SA, were characterized by a reduced level of pathogen-induced proline accumulation. It should be noted that, under the influence of bacilli in healthy stored tubers, a slight decrease in the amount of proline was also observed, which additionally indicates the important role of this agent in the formation of induced resistance to the causative agent of fusariosis.

Interestingly, based on the results in Figure 1, SA with 26D has a negative effect when compared with only 26D and other treatments upon *F. oxysporum* infection. However, the degree of MDA and proline accumulation is lower than in variants with other treatments. This can be in the case of either blocking the formation of these substances in the plant organism or digesting data compounds with pathocomplex *F. oxysporum* and 26D in the presence of SA. Perhaps, this is the so-called cross-talk in metabolism, when some effectors interfere with the work of others and the level of the protective barrier decreases, while the phytopathogen gets the opportunity to develop. Probably, this might also be connected with the ability of exogenous SA (when applied together with 26D) to induce other pathways that lead to cell strengthening in the initial stages of storage and tubers by six months of storage are characterized by a lower degree of cell damages. Additionally, it might be connected with the ability of 26D produce compounds that is not comparable with SA (for example, through JA production and inducing JA-depending signaling pathways); or perhaps if 26D resulted in the production of endogenous SA in tubers in high concentrations and additional exogenous SA resulted in “feedback mechanism”, when the formation of high levels of this compound (along with the additional introduction of exogenous SA) can lead either to the neutralization of their positive effect or even to a negative effect on disease development. These assumptions, of course, require close attention and further detailed investigations.

A biochemical marker for the formation of resistance can also serve the data about the changes in reducing sugar (RS) content in healthy and pathogen-infected tubers during storage [31,32]. The results showed that, in healthy stored potato tubers, *B. subtilis* and *B. subtilis* + SA decreased the RS content (up to 1.2–2.5 times) in comparison with the control (Figure 5), which indicated that *B. subtilis* also feed on sugars, including those that result from pathogen-induced activation of hydrolytic enzymes that break down starch into sugars. It was suggested that discovered changes in RS in *B. subtilis* and SA treated potato tubers upon fusarium infection may be due to the pathogens during the attack secrete hydrolytic enzymes, under the influence of which starch breaks down into sugar in the cells of affected tubers, including reducing starch, which is later used by phytopathogens as one of the most available power supplies. Interestingly, in healthy stored tubers *B. subtilis* and *B. subtilis* + SA decreased RS content (up to 1.2–2.5 times) in comparison with the control (Figure 5), which indicated that *B. subtilis* also feed on sugars, including those that result from pathogen-induced activation of hydrolytic enzymes that break down starch into sugars. These data indicate that *B. subtilis* bacteria effectively compete with pathogens for accessible food sources, in particular sugars, and generally create an unfavorable environment for the further development of pathogens inside tubers, which inevitably leads to the inhibition of their growth and, as a result, a decrease in the incidence of tubers during storage. It is likely endophytic *B. subtilis* can themselves destroy starch to sugars and/or feed on existing sugars, including those that result from pathogen-induced activation of hydrolytic enzymes that break down starch to sugars. Moreover, *B. subtilis* are capable of producing compounds with antibiotic activity and inhibit the propagation of pathogens, compete for available food sources and, in general, create an unfavorable environment for the further development of pathogens inside tubers [9], which inevitably leads to the inhibition of their growth and how a consequence of a decrease in the incidence of tubers during storage, and the remaining RS can be spent on food by bacteria. Changes in the content of RS in healthy and *Fusarium*-infected tubers during storage can serve not only as an important biochemical marker for the formation of resistance [32], but can also determine the consumer qualities of stored potatoes [31]. An increase in the RS content in potato tubers during storage negatively affects the culinary qualities (during heat treatment the flesh darkens, the taste worsens and becomes sweet-starchy-viscous). The findings on a decrease in the content of RS in healthy and *Fusarium*-infected tubers under the influence of *B. subtilis* and *B. subtilis* + SA indicate that they positively affect the consumer qualities of stored tubers by this indicator.

Other important indicators, which largely determine the consumer and table qualities of potatoes, are the content of starch and total dry matter (TDM) in tubers [31], which can be a judge for the best further options for processing products. The high content of TDM in tubers is a criterion for the low absorption capacity of oil during potato frying [43]. According to the criterion of starch content, potatoes are divided into 1) starchy (or powdery) (with a high starch content, an average of about 22%), which, when cooked, acquires a granular texture, which is optimal for deep fat, and is also suitable for making mashed potatoes and for baking; 2) wax (potatoes with a lower starch content, on average about 16% and below), which is suitable for cooking and salads, because the tubers maintain the integrity of the fabric. The starch content can vary greatly based on the varietal characteristics of potatoes, the place of growth, climatic conditions, and other factors. At the same time, the process of starch decomposition into sugar during storage, which gives the tubers a sweet taste and negatively affects the taste, is of particular importance. In general, during storage, the decomposition of starch in potato tubers can occur by hydrolytic (with the participation of amylase) and phosphorylithic (with the participation of phosphorylase) pathways. In healthy tubers, amylase activity is often not detectable or is very low. However, the infestation of tubers with phytopathogens and various types of microorganisms, including bacteria, leads to an increase in the activity of hydrolytic enzymes and, accordingly, to an increase in the level of starch decomposition [44]. At the same time, potato starch granules can be quite resistant to destructive enzymes [43]. However, as the tubers germinate, the starch granules decompose, which indicates the formation of destructive enzymes into tubers. Some authors reported that *Bacillus circulans* capable of breaking potato starch granules [45] and some bacteria, including *B. subtilis*, *B. amyloliquefaciens*, and *Microbacterium aurum*, are capable of producing AMY [34,44,46,47]. Storage at 3 °C also increases the activity of AMY in potato tubers, however, an increase can also occur due to the general reaction to stress in tubers, which coincides with cold sweetening, and the enzyme does not directly participate in starch degradation [44]. Despite current information, the biological process of starch degradation in potato tubers remains unclear [44,45] and information on the effect of endophytic *B. subtilis* bacteria and their compositions with SA on starch content in healthy and fusarium-infected tubers during long-term storage is not available in the available literature. Our results showed that infestation by *F. oxysporum* results in the reduction of starch content in stored potatoes from 14.4% (in non-infected control) to 6.2% (in infected tubers) (Figure 6A). The application of 26D, 26D + SA, and 10-4 + SA prevented such pathogen-affected starch reduction with maximum positive effects for 26D and 26D + SA. In healthy tubers, 10–4, 26D, and 26D + SA decreased the starch level, while, for 10-4 + SA, a slight increase was observed. In general, these data indicate a positive effect of *B. subtilis* and SA on starch safety in tubers that were infected with pathogens during storage. Additionally, the phenomenon of a slight decrease in starch in healthy tubers (not infected with pathogens) under the influence of bacterial treatments might be because *B. subtilis* bacteria may use starch as a substrate for nutrition and/or destroy starch due to the production of hydrolytic enzymes. The same pattern of change from *B. subtilis* (10-4, 26D) with and without SA were observed for total dry matter (TDM) levels in non-infected (healthy) and *Fusarium*-infected tubers (Figure 6B). In healthy tubers treated with 10-4, 26D, 26D + SA after long-time storage the level of TDM concentration decreased (up to 16.1–18.1%) in comparison with the control, while 10-4 + SA caused an increase (Figure 6B). The increased levels of starch and TDM percentages in healthy tubers that are treated with *B. subtilis* 10-4 + SA (Figure 5A,B) are most likely to happen due to the trigger of some other additional protective mechanisms by SA in composition with 10-4, while, for the composition 26D + SA the picture was different. Additionally, the phenomenon of a slight decrease in starch in healthy tubers (not infected with pathogens) under the influence of bacterial treatments might be due to the fact that *B. subtilis* bacteria use starch as a substrate for nutrition and/or destroy starch due to the production of hydrolytic enzymes. Indeed, it was found that, in healthy stored tubers, under the influence of treatments with the tested strains 10-4, 26D, an increase in AMY activity occurred (Figure 3B), which is also consistent with the available published data on the ability of a number of non-pathogenic bacteria, including *Bacillus* spp., in order to produce AMY [9,34,44,46,47]. Earlier, we also demonstrated the ability of the studied bacteria 10-4 and 26D to colonize the internal tissues of both healthy and infected tubers, thereby preventing the spread of the pathogen and reducing the incidence of tubers [16]. At the same time, in healthy tubers, the highest starch content was found for treatment 10-4 + SA (Figure 6A). This indicates that the composition 10-4 + SA helps to inhibit the decomposition of starch in tubers during storage. A similar picture was obtained when assessing the changes in the TDM in healthy and *F. oxysporum*-infected tubers under the influence of tested treatments (Figure 6B). Most likely, this nature of the action of strain 10-4 with SA can be a consequence of the fact that SA in compositions with strain 10-4 acts as a trigger of some protective mechanisms, while the picture is different for composition 26D + SA. The revealed difference in the action of the tested strains, both individually and in compositions with SA, can be largely related to the ability of 10-4 and 26D to produce a wide range of protective compounds, including their own phytohormones or other biologically active substances [9,11]. Strain 10-4 likely produces SA, and the addition of exogenous SA can enhance its protective effect; if strain 26D produces jasmonic acid (JA), the addition of SA to the composition might have the opposite effect, since JA and SA are antagonists [9]. This hypothesis, of course, requires additional studies to verify. With that, the results of our analysis allow for us to suggest that *B. subtilis* and SA in general impact positive influence in preserving starch and TDM contents in *Fusarium*-infected tubers indicating their higher resistance. These findings are consistent with data from the literature regarding tubers, with higher TDM and starch concentrations exhibiting the highest resistance to mechanical impacts [34]. It might be assumed that the reason for the increased resistance of tubers with a higher concentration of starch and TDM to stresses is associated with a certain cell structure of these tubers; therefore, significant force is required to damage the cell structures.

As a result of exposure to different stress factors (light, injuries, microorganisms, extreme temperatures, etc.), normal metabolic pathways change in potato, which lead to the synthesis of abnormal amounts of numerous novel compounds and secondary metabolites, including toxic glycoalkaloids (GA), at levels that exceed the amount found in healthy tissues, also accompanied by the synthesis of other not usually found in the absence of stress [38]. An important role in the natural disease resistance of tubers is played by GA (in particular, steroid glycosides α-Solanine, and α-Chaconine), being concentrated mainly in their integumentary tissues [37]. However, GA is also potentially health-threatening toxic compounds in potatoes that pose a serious potential hazard to humans [38,39]. The most toxic among GAs of potato are α-Chaconine and α-Solanine, which comprise approximately 95% of the total GA (TGA). Elevated levels of GA (200 mg/kg of tubers are considered to be critical) in potato have a toxic effect on the nervous and gastrointestinal systems of humans [38]. The literature describes many specific examples of poisoning (sometimes lethal) of people as a result of eating potatoes with a high GA content [48]. According to some reports, GA content od higher than 100 mg/kg fresh weight (FW) may lead to a bitter flavor in potatoes [38]. In addition, GA accumulation is associated with the greening of tubers and light-induced processes [49], but a causal relationship between the two processes has not been established [50]. The greening of tubers occurs due to the formation of chlorophyll, which is a useful non-toxic indicator that tubers have been exposed to light and should no longer be consumed [49]. However, GA formation can also occur in the non-green parts of tubers. Therefore, GA formation and the greening of potatoes are physiologically unrelated processes [51]. The formation of GA can be influenced by many factors, including genotype, growing conditions, transportation, storage, temperature, germination, exposure to light, phytopathogens, herbicides, biostimulants, etc. [38,51]. However, the highest accumulation of toxic levels of GA in potatoes typically occurs in the postharvest storage period. Although the mechanisms of action of GA in potatoes are not completely clear, it is believed that they are associated with antimicrobial and antifungal activities [35,36]. Several authors have reported an increase in GA content in potato in response to stresses of various nature and its significance as an important factor for plant survival in extreme situations. In general, it is believed that steroidal alkaloids, being secondary metabolites of plants [52], confer resistance to microbial diseases. However, the correlation between GA content in potato tubers during storage and under conditions of infection by the causative agents of Fusarium dry rot desease, and the use of endophytic bacteria B. subtilis and B. subtilis + SA in the available literature has not been established at the moment of the beginning of our work. Our experiments showed that the infection of tubers with F. oxysporum caused an increase in the level of GA, both α-Solanine and α-Chaconine by the sixth month of storage (Figure 6A,B). The application of B. subtilis (10-4, 26D), individually and in compositions with SA, significantly decreased such pathogen-induced elevated levels of α-Solanine and α-Chaconine, thereby indicating their involvement in the development of tubers resistance and the positive impact of the studied bacteria on the quality of stored products. Interestingly, in non-infected (healthy) tubers the content of α-Solanine and α-Chaconine had a different profile, depending on the variant of treatment (Figure 6A,B). Accordingly, in variants with the application of 10-4 and 26D + SA, there was no significant difference from control in the content of α-Solanine and α-Chaconine. In contrast, the application of 10-4 + SA increased (up to 2–2.3 times in comprison with control) both α-Solanine and α-Chaconine contents in healthy tubers. Additionally, the application of 26D resulted in decreased (up to 1.4 times) α-Solanine and α-Chaconine in comparison to the control. These results suggest that B. subtilis, in compositions with and without SA, regulate GA content in stored healthy and Fusarium-infected tubers. However, the joint application of B. subtilis with SA changes GA content (α-Solanine, α-Chaconine) differently in a strain-dependent manner indicating that studied strains (10-4, 26D) use different protective mechanisms, especially when they are applied in combination with SA.

## 4. Materials and Methods

### 4.1. Bacterial Strains and Phytopathogenic Fungi

The endophytic bacteria *B. subtilis* 10-4 was previously isolated from arable soils of the Republic of Bashkortostan (Russia) at the Bashkir Research Institute of Agriculture UFRC RAS (Ufa, Russia) and described [53]. The endophytic bacteria *B. subtilis* 26D (the basis of the commercial biological product Fitosporin-M, BashInkom S&IE, Ltd, Ufa, Russia) was kindly provided by the microbiological laboratory of BashInkom S&IE, Ltd (Ufa, Russia). *B. subtilis* (strains 10-4 and 26D) cells were cultured on potato-glucose agar (PGA) medium at 37 °C for 3–4 days [53,54]. To obtain inoculums of strains 10-4 and 26D were prepared the suspensions containing 10^8^ colony forming units (CFU)/mL of bacteria according to 0.5 McFarland Turbidity Standard (were additionally monitored by the optical density at 600 nm (OD600) (SmartSpecTM Plus spectrophotometer, Bio-Rad, Hercules, CA, USA)), and then diluted down to 10^7^ and 10^6^ CFU/mL while using distilled water or/and solutions of SA (0.05 mM) in distilled water. *F. oxysporum* (causative agent of Fusarium dry rot) was obtained from the collection of microorganisms of the Laboratory of Plant-Microbe Interactions of the Bashkir Research Institute of Agriculture UFRC RAS (Ufa, Russia) and the Microbiological Laboratory of the BashInkom S&IE, Ltd (Ufa, Russia). The phytopathogenic fungus *F. oxysporum* was grown on PGA (pH 6.6) at a temperature of 28 °C [54]. The concentrations of the phytopathogenic fungus *F. oxysporum* (10^6^ spores/mL) were prepared while using a Goryaev chamber [54].

### 4.2. Plant Materials and Potato Growth Conditions

The experiments were carried out on hydroponically grown potato (*Solanum tuberosum* L., Cv. Bashkirsky) mini-tubers provided by the Laboratory of Potato Breeding and Seed Production of the Bashkir Research Institute of Agriculture UFRC RAS (Ufa, Russia) [16]. Cv. Bashkirsky was created by breeders of the Bashkir Research Institute of Agriculture (Ufa, Russia) and All-Russian Research Institute of Potato of A.G. Lorch (Moscow, Russia) from botanical seeds that were obtained in 1991 when crossing Cv. Belousovsky and hybrid 289/82-3 from All-Russian Research Institute of Potato of A.G. Lorch (Moscow, Russia). Cv. Bashkirsky was included in the State Register of Breeding Achievements in 2007 for the Ural region. Cv. Bashkirsky characterized as resistant to the causative agent of potato cancer, being slightly affected by the golden potato cyst-forming nematode and resistant to drought. A feature of the Cv. Bashkirsky is the property of "dropping" the egg-laying of the Colorado potato beetle from itself, since the leaves of this plant have a hypersensitive reaction, and it independently cleans itself of pests. It has a high necrogenetic and inhibitory barrier [55].

In vitro plants of healthy potatoes were placed in hydroponic equipment (“Minivit”, KD-10, Russia) and grown in a continuously supplied nutrient solution of a commercial complex water-soluble fertilizer with microelements Novalon (19-19-19 + 2 MgO + ME, Novalon, DoctorTarsa, Turkey) (pH 5.6 mol L) with a concentration of 0.4–0.6% (for one week), 0.8% (for two weeks), 1.2–1.4% (for three weeks), and 1.5–1.8 % (from four weeks to the end of the growing season). The obtained (freshly harvested) hydroponic mini-tubers of potato were characterized by an oval-rounded shape with medium-depth eyes, smooth red skin, and white flesh. The biomass of hydroponically grown mini-tubers was 4–6 g. The growing season was about 60–70 days. The lighting mode was divided into three main periods and amounted to 120,000, 150,000, and 80,000 lux/h, respectively. The detailed scheme of the experiment was presented in our previous paper [16].

### 4.3. Scheme of the Experiment and Storage Conditions

Immediately before laying to storage, freshly harvested hydroponic mini-tubers (4–6 grams/tuber) of potato were infected by *F. oxysporum* (10^6^ spores/mL) by immersion into the pathogen solution for 30 min (control (non-infected or «Healthy») tubers were immersed into water), hereafter the pathogen solution and water was drained and the tubers were air-dried (20–30 min at room temperature). Subsequently, the dried tubers were immersed for 30 min in solutions of water (control), *B. subtilis* (10-4 and 26D) (10^8^ CFU/mL), *B. subtilis* 10-4 (10^7^ CFU/mL) + SA (0.05 mM), and *B. subtilis* 26D (10^6^ CFU/mL) + SA (0.05 mM) in previously selected concentrations effectively reducing pathogen development [16]. Afterwards, the solutions were also drained, the tubers air-dried (20–30 min at room temperature) and stored (thermostat TVL-K 120, INSOVT, Russia) at a temperature of 18 ± 1 °C for two weeks (treatment period) and then 3 ± 1 °C for six months to record disease incidence and assess the physio-biochemical and quality parameters [16]. Each treatment was conducted in triplicate with 30 samples per replicate.

### 4.4. Antagonistic Assays of Antifungal Activities

Qualitative bioassay for anti-fungal potency of *B. subtilis* 10-4 and 26D was done using the co-culture of bacterial strains and indicator fungus *F. oxysporum* previously plated on potato-dextrose (PDA) medium [54]. An agar well diffusion assay was done to test the antifungal potency. An aliquot of 150 µL of bacterial culture medium was filled in a well of 5 mm diameter made in PDA (pH 7.0) previously plated with a spore suspension of the fungus. The incubation at 30 °C for 3 days was followed by clearance zone estimation due to fungal growth inhibition. At least three replicates were done for each experiment. The visualization of pathogen mycelium growth was imaged using a scanning electron microscope Biozero BZ-8100E (Keyence Co., Osaka, Japan).

### 4.5. Assessment F. Oxysporum—Caused Fusarium Dry Rot Development in Stored Tubers

Visual symptoms of diseases development were evaluated on a five-point scale (0 points—no symptoms, 1 point—damage from 1 to 25%, 2 points—from 26 to 50%, 3 points—from 51 to 75%, 4 points—more than 75%; 5 points—100% completely affected). An analysis of the intensity of the development of diseases on tuber slices was evaluated according to [16].

### 4.6. Determination the Activity of Hydrolytic Enzymes Amylases (AMY), Proteinases (PRO) and Their Inhibitors

The activity of AMY, PRO, and their inhibitors was determined according to [56,57]. A sample of tubers was homogenized in a porcelain mortar with glass sand and resuspended in a single volume of buffer solution (0.05 M Tris-HCl (pH 8) for proteases; 0.2 M phosphate buffer (pH 6) for amylases) and kept for 30 min at 4 °C. The obtained extracts were centrifuged twice at 10,000 rpm for 10 min at 4 °C (5417R centrifuge, Eppendorf AG, Hamburg, Germany). The supernatant was used as a source of proteolytic, amylolytic, and inhibitory activity. Supernatants were incubated with a standard enzyme solution to determine *Bacillus subtilis* AMY inhibitors and trypsin inhibitors (1:1 volume ratio). Solutions of enzymes in distilled water were used as a control. For the preparation of standard enzyme solutions, commercial preparations of AMY *Bacillus subtilis* (Sigma, USA) and trypsin (Sigma, USA) was used. A portion of the *Bacillus subtilis* AMY preparation was dissolved in phosphate buffer and trypsin in Tris HCl buffer (100 µg/mL). Gelatin and starch were used as a substrate for tuber PRO and AMY, respectively.

The determination of enzymatic and inhibitory activities was carried out by hydrolysis of a substrate immobilized in a polyacrylamide gel plate [56,57]. During incubation, the enzyme molecules from the solution diffuse into the gel and the substrate is hydrolyzed. Treatment with dye after incubation revealed areas of the gel with a hydrolyzed substrate. The enzyme activity was expressed in arbitrary units that were obtained by digital processing of the stained areas of the gel using an original Software [58]. The activity of the inhibitor was calculated by the difference in the activity of the free enzyme and the mixture of the enzyme and the inhibitor. For one conditional unit of inhibitor activity (mIE), an amount of inhibitor was taken that suppressed one conditional ME of free enzyme activity.

### 4.7. Assessment of Glycoalkaloids (GA) (total GA [TGA], α-Solanine, α-Chaconine) Content

GA (α-Solanine, α-Chaconine, TGA) content was determined using the HPLC method [59]. Samples (10 g) of diced tubers were homogenized in 40 mL extraction solution (water:acetic acid:sodium hydrogen sulfide, 100:5:0.5, *v/v/w*) for 15 min in an Ultra Turrax T-50 homogenizer (Daigger Scientific, Vernon Hills, IL, USA) and then centrifuged at 3000 rpm for 10 min, following which the supernatant was collected and stored at + 4 °C in the dark for up to 24 h. Subsequently, the extract was cleaned by passage through SPE columns (Sep-Pak C18: silica-based octadecyl bonded phase with strong hydrophobicity, 500 mg sorbent per cartridge, particle size 55–105 µm; Waters Corp., Milford MA, USA) that were first conditioned with 5 mL acetonitrile and then with 5 mL extraction solution. Samples of extracts (10 mL) were passed through the column, washed with 4 mL of 15% acetonitrile, and then eluted with 4 mL of mobile phase; the volume was then adjusted to 5 mL with the mobile phase [59]. Thereafter, 20 µL of the sample or the standard solution was injected, GA was decomposed using isocratic elution with 50% acetonitrile in an HPLC system that was equipped with a C18 Atlantis column (5 µm, 3.9 mm × 150 mm; Waters Corp., Milford MA, USA) at a flow rate of 1.5 mL/min. and 0.01 mol/L phosphate buffer (10% 0.1 mol/L, pH 7.6). The results were obtained by measuring at 202 nm with a UV-detector and by comparing the regions covered by the standards of α-Solanine and α-Chaconine. TGA content was calculated as the sum of α-Solanine and α-Chaconine [59] in milligrams GA per kilogram fresh weight.

### 4.8. Estimation of Lipid Peroxidation (MDA) and Proline (Pro) Content in Tubers

The MDA concentration was estimated according to the method of Health and Packer [60]. Fresh tubers (0.2 g) were homogenized with 10% trichloroacetic acid (1 mL) and centrifuged at 10,000 rpm for 10 min. Thereafter, 1 mL of the supernatant was mixed with 20% trichloroacetic acid containing 0.25% thiobarbituric acid and was heated at 95 °C for 30 min., quickly cooled in an ice bath, and then centrifuged again at 10,000 rpm for 10 min. The absorbance of the supernatant was read at 532 and 600 nm (SmartSpecTM Plus spectrophotometer, Bio-Rad, Hercules, CA, USA). The MDA concentration was calculated using an extinction coefficient of 155 mM^−1^cm^−1^ and expressed as nmol g^−1^ FW.

Pro was determined according to Bates et al. [61]. Fresh tissue of stored tubers (0.5 g) was extracted with 2.5 mL of boiled water. Subsequently, 2.0 mL extract was mixed with an equal volume of ninhydrin solution (1.25 g ninhydrin dissolved in 30 mL glacial acetic acid, and 20 mL of 6 M phosphoric acid) and glacial acetic acid. The samples were then incubated at 100 °C for 1 h and the reaction was terminated by cooling the tubes in an ice bath. After cooling, Pro was determined at 522 nm (SmartSpecTM Plus spectrophotometer, Bio-Rad, Hercules, CA, USA).

### 4.9. Assessment of Reducing Sugars (RS), Starch, and Total Dry Matter (TDM) Contents in Tubers

The reducing sugars were accessed using Samner’s reagent. The starch content and TDM in fresh tubers were calculated according to using a specific weight of potato tubers to quantity both in air and after their immersion in a water glass [62,63].

### 4.10. Statistical Analysis

All of the microbiological, biochemical, and physiological experiments were performed at least three biological and three analytical replicates. The data were presented as the mean ± standard error (SEM). Statistically significant differences between the mean values were evaluated using a two-way analysis of variance (ANOVA), followed by the Tukey test (*p* < 0.05).

## 5. Conclusions

In summary, the results that are presented here establish that the treatment of potato tubers immediately before storage with endophytic bacteria *B. subtilis* (10-4, 26D) individually and in combinations with SA reduced the incidence of *F. oxysporum*-mediated dry rot (up to 50%) in potatoes during long-term storage, with the highest protective effect upon application of composition *B. subtilis* 10-4 + SA. The obtained results support an argument in favor of the protection of cells from the damaging effect of *F. oxysporum* and control of aging processes under the influence of *B. subtilis* and *B. subtilis* + SA due to the ability of these bacteria to destroy the cell wall of pathogens as well as to activate hydrolytic enzyme AMY and AMY inhibitors. Additionally, the application of *B. subtilis* and *B. subtilis* + SA resulted in decreased *Fusarium*-induced lipid peroxidation, proline, reducing sugar, and glycoalkaloids (α-Solanine and α-Chaconine) accumulation and contribute to preserving the increased level of starch and total dry matter in stored potatoes upon the infection of *F. oxysporum*. These findings confirm, on the one hand, the involvement of studied compounds in cell protections against *F. oxysporum*, and, on another hand, the positive influence of bacteria *B. subtilis* (10-4, 26D) and *B. subtilis* (10-4, 26D) + SA on the quality of stored tubers. With that, depending on bacterial strain, their effect on stored tubers after combined use with SA might vary (SA notably enhanced the positive effect of strain 10-4, while for 26D such effect was not observed), indicating on mechanisms of interactions of *B. subtilis* strains with SA is complicated, intertwined, and require careful attention and further investigations. In general, the obtained data indicate in favor that the application of endophytic bacteria *B. subtilis* individually and in combinations with SA is a promising effective eco-friendly and bio-safe strategy to cope with postharvest decays of stored potato; however, when developing preparations-compositions should take into account the strain-dependent manner of *B. subtilis* action together with SA.
